# Efficient Coupled-Cluster Python Frameworks for Next-Generation GPUs: A Comparative Study of CuPy and PyTorch on the Hopper and Grace Hopper Architecture

**DOI:** 10.1021/acs.jctc.6c00558

**Published:** 2026-07-02

**Authors:** Antonina Dobrowolska, Julian Świerczyński, Paweł Tecmer, Emil Sujkowski, Somayeh Ahmadkhani, Grzegorz Mazur, Klemens Noga, Jeff Hammond, Katharina Boguslawski

**Affiliations:** † Institute of Physics, Faculty of Physics, Astronomy, and Informatics, 49577 Nicolaus Copernicus University in Toruń, Grudziadzka 5, Toruń 87-100, Poland; ‡ Department of Computational Methods in Chemistry, Jagiellonian University, Faculty of Chemistry, Jagiellonian University, Gronostajowa 2, Kraków 30-387, Poland; § Academic Computer Centre Cyfronet AGH, Nawojki 11a, Kraków 30-950, Poland; ∥ NVIDIA Helsinki Oy, Helsinki 00180, Finland

## Abstract

In this work, we introduce new batching algorithms to effectively handle large contractions encountered in coupled-cluster singles and doubles (CCSD) implementations in Python on the video random access memory (VRAM) of graphical processing units (GPUs), thereby improving performance. Specifically, we benchmark the performance of the CuPy and PyTorch libraries on a single NVIDIA Hopper (H100) and the Grace Hopper (GH200) architectures. We begin by optimizing the particle–particle ladder bottleneck contraction in CCSD using an asymmetric and dynamic splitting recipe, and then move toward a generic tensor contraction protocol that enables tensor contractions to be performed almost exclusively on GPUs. We benchmark our new, fully generic GPU-accelerated coupled-cluster implementations for various molecular systems and basis-set sizes, using both the CuPy and PyTorch libraries. While PyTorch outperforms CuPy on H100 by approximately 20%, both perform similarly on the GH200 architecture. Compared to our initial GPU implementation [J. Chem. Theory Comput. 2024, 20, 3, 1130–1142], we achieve a 10-fold speedup. In molecular CCSD calculations, we report additional speedups between 3 and 16 for a single CCSD iteration using Cholesky-decomposed electron repulsion integrals compared to our original GPU-CPU hybrid implementation.

## Graphical abstract

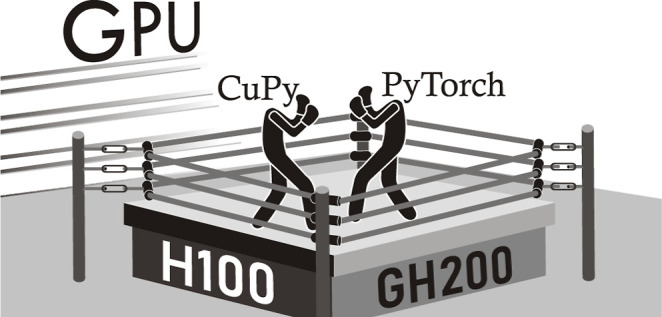

## 1 Introduction

Rapid advances in modern graphics processing unit (GPU) hardware have fundamentally transformed scientific computing over the past two decades, enabling unprecedented scale and speed in simulations across diverse domains of computer-aided research. These include, among others, molecular dynamics, climate modeling, drug discovery, astrophysics, materials science, and quantum chemistry, where GPUs facilitate the modeling of complex systems with orders-of-magnitude improvements in throughput compared to traditional central processing unit (CPU)-based approaches.^1–6^ At the core of this revolution is the GPU’s massively parallel architecture, which efficiently processes large volumes of data by executing independent tasks concurrently on thousands of cores. The GPU architecture differs fundamentally from that of CPUs, which typically feature only tens of cores and are optimized for sequential processing and complex control-flow workloads. Another key difference lies in the memory hierarchy. CPUs rely on general-purpose system RAM (random access memory), which prioritizes low latency and versatility across a wide range of tasks. GPUs, in contrast, employ dedicated high-bandwidth VRAM, engineered for rapid, high-throughput parallel access in graphics rendering and compute-intensive workloads. As a result, when algorithms exhibit high data parallelism, GPUs can deliver speedups of 10 to 100 times over multicore CPUs, dramatically reducing time-to-solution and energy consumption.^7–10^

In quantum chemistry, GPUs excel at computing two-electron repulsion integrals (ERIs),^11–14^ and effective core potentials,^15^ performing density functional theory (DFT)^8,16,17^ and localized active-space self-consistent field (LASSCF) calculations,^18^ and efficiently constructing the Fock matrix—a major computational bottleneck in self-consistent field (SCF) procedures.^8,17,19–23^ However, the use of GPUs for large-scale correlated post-Hartree–Fock electronic structure calculations—such as those based on the coupled-cluster (CC) ansatz—remains limited. Yet, harnessing the full potential of GPUs may require a serious rewrite of complex electronic structure codes. Hence, modular, library-heavy electronic structure implementations are desirable, where GPU-accelerated computing is accessible through libraries, and computations on CPUs and GPUs are dynamically accessible via interfaces.^24–27^

As an example, Python offers several open-source libraries^28–30^ for both CPU^31^ and GPU-accelerated computing.^25^ To this end, the rapid development of GPU hardware architectures has led to an immediate growth in Python-based software packages that enable convenient migration of CPU-oriented code to GPUs without requiring low-level programming, making it highly compatible with the NumPy library.^31^ Examples are CuPy,^29^ PyTorch,^30^ and TensorFlow.^28^ Although the main driving force behind the development of GPU-supported Python libraries is machine learning, these libraries become practical in quantum chemistry-oriented problems.^9,10,25,32,33^ Recently, we explored a Python-based coupled cluster singles and doubles (CCSD) implementation using Cholesky-decomposed^34,35^ two-electron integrals in the PyBEST software package^36,37^ using the CuPy library.^25^ We showed that to work within the 32 GB of VRAM on the NVIDIA Tesla V100S PCIe GPU, tensor contractions must be executed in a batch-wise manner, dividing large intermediate tensors into smaller subproblems that fit available device VRAM. This batching strategy was carefully tuned for the V100S architecture to maximize efficiency. Despite this memory constraint, offloading the key tensor contractions to the GPU using CuPy yielded a speedup of 10–16 times for the dominant bottleneck contraction relative to an equivalent NumPy-based implementation running on 36 CPU cores.^25^

A solution to the limited VRAM problem on single GPUs, such as the V100S, lies in newer architectures like NVIDIA’s Hopper-based systems^24^ and the Grace Hopper Superchip (GH200), which additionally offer substantially increased high-bandwidth memory (HBM).^38^ A single Hopper GPU in the GH200 provides up to 96 GB of HBM3. Beyond expanded dedicated GPU memory, the GH200 is a true heterogeneous superchip that tightly integrates an Arm-based Grace CPU (72 cores) with the Hopper GPU via the high-speed NVLink-C2C interconnect. This coherent, memory-unified design enables low-latency, cache-coherent data sharing between the CPU’s system memory and GPU HBM. As a result, traditional PCIe data-transfer bottlenecks are largely eliminated. More computational advantage is to be offered within NVIDIA’s Blackwell architecture,^39^ particularly when exploiting FP64 emulation,^40–42^ which has been previously demonstrated for the DGEMM-intensive density-matrix renormalization group (DMRG) method^43^ and the density-matrix purification method for solving the self-consistent field (SCF) equations.^44^ All together, this motivates us to further develop and optimize Python-based CC implementations specifically for these newer architectures. In this paper, we propose and extensively test alternative GPU batching algorithms against the CuPy and PyTorch libraries for various system sizes and contraction types on single H100 and GH200 architectures. Most importantly, these new batching approaches can bring us closer to native CUDA implementations for CCSD.

This work is organized as follows: In Section 2, we discuss the main bottleneck operations in CC calculations. Section 3 describes algorithmic techniques designed to maximize GPU memory efficiency. Section 4 lists computational details. Numerical results and the assessment of the GPU to CPU performance are presented in Section 5. Comparisons with other Python-based developments of CC codes are discussed in Section 6. Finally, we conclude and provide an outlook in Section 7.

## 2 Acceleration of CC Calculations

In this work, we build upon our previous GPU-accelerated Pythonic CC implementation.^25^ Specifically, we focus on the CC ansatz^45–50^

1 $$|\Psi \rangle =e^{\mathrm{T̂}}|\Phi_{0}\rangle$$

where the cluster operator T̂ is restricted to, at most, double excitations, that is, $\mathrm{T̂}={\mathrm{T̂}}_{2}$ or $\mathrm{T̂}={\mathrm{T̂}}_{1}+{\mathrm{T̂}}_{2}$ (|Φ_0_⟩ is some reference wave function like the Hartree–Fock determinant). Note, however, that the bottleneck operations of CCSD are due to the ${\mathrm{T̂}}_{2}$ excitation operator. Thus, we will focus our discussion on ${\mathrm{T̂}}_{2}$ -related terms in the CC working equations. Furthermore, we will consider a spin-free representation, where the amplitudes bear no information about spin degrees of freedom and the CC equations are spin-summed. The spin-free double excitation operator takes on the form

2 $${\mathrm{T̂}}_{2}=\frac{1}{2}\sum_{ij}^{o}\sum_{ab}^{v}t_{ij}^{ab}{Ê}_{ai}{Ê}_{bj}$$

with the CCD amplitude *t*_*ij*_^*ab*^ and ${Ê}_{ai}$ being the singlet excitation operator, running over all occupied (o) and virtual (v) orbitals for the chosen reference determinant |Φ_0_⟩

3 $${Ê}_{ai}={â}^{†}î+{\hat{\overset{®}{a}}}^{†}\hat{\overset{®}{i}}$$

where ${â}^{†}$ $\left({\hat{\overset{®}{a}}}^{†}\right)$ labels electron creation operators for α (β) electrons, while î $\left(\hat{\overset{®}{i}}\right)$ indicates the corresponding annihilation operators.

The scaling-determining step in the CCD amplitude equations is the so-called particle–particle ladder term

$$0=...+\underset{cd}{\sum }\langle ab|cd\rangle t_{ij}^{cd}+...$$ 4

where a summation over the indices *i*, *j*, *a*, *b* is implied and ⟨*ab*|*cd*⟩ are the electron-repulsion integrals in physicist’s notation. This term determines the well-known formal scaling of the CCD (or CCSD) equations of $O\left(o^{2}v^{4}\right)$ . In large-scale modeling, however, full storage of the electron-repulsion integrals (ERI) is typically prohibitive. As a remedy to save storage, approximate ERIs are used. One possibility is to exploit Cholesky decomposition of the ERI^34,51^

$$\langle ab|cd\rangle \approx \underset{x}{\sum }L_{ac}^{x}L_{bd}^{x}$$ 5

where *x* indicates the summation over the elements of the Cholesky vectors. The dimension of *x* depends on the chosen threshold of the Cholesky decomposition. For decent to tight thresholds (around 10^–5^), we typically have *x* ≈ 5­(*o* + *v*). Since we work with real restricted orbitals that result in 8-fold permutational symmetry of the ERI, both Cholesky vectors *L*_*ac*_^*x*^ and *L*_*bd*_^*x*^ are identical and only one vector needs to be stored. Using the approximate Cholesky decomposition of the ERI in eq 5, we can rewrite the CCD (or CCSD) amplitude equations in eq 4 so that the *n*-th iteration of the CC vector function t̃ijab(n) evaluation becomes (again focusing on the particle–particle ladder term only)

t̃ijab(n)=...+∑xcdLacxLbdxtijcd+... 6

A naïve summation over the Cholesky vectors may give the impression that the computational complexity increases to $O\left(xo^{2}v^{4}\right)$ . By defining suitable intermediates, the formal scaling can be, however, reduced to $O\left(xo^{2}v^{3}\right)$ (if *x* > *o* ^2^, creating an intermediate array of size *xo* ^2^ *v* ^2^) or $O\left(o^{2}v^{4}\right)$ (if *v* > *o* ^2^, creating an intermediate array of size *v* ^4^), respectively. In our previous work,^25^ we GPU-accelerated the evaluation of the Cholesky-decomposed particle–particle ladder term using the latter path, formally generating an intermediate of size *v* ^4^. In order to store all intermediates on the VRAM, we proposed a batching recipe in which we split the axes *x*, *a*, *b*, and, if needed, *i*, and perform tensor contraction operations for the subblocks in question. Overall, by offloading the bottleneck contraction of the CCD/CCSD vector function evaluation, we achieved a speed-up factor of 3–4 within the CuPy library compared to the CPU-only implementation (in Python using various NumPy methods, see also ref 25 for details).

In the following, we will use the numpy.einsum subscript convention to label tensor contraction operations. For instance, the term on the right-hand side of eq 6 can be translated as the “xac,xbd,icjd→iajb” subscript. Furthermore, for reasons of generality, we will not discriminate the Cholesky-decomposed ERI from their dense representation when dealing with the formal subscript notation of the underlying tensor contraction operation. Hence, the terms on the right-hand side of eqs 4 and 6 will be labeled using the “abcd,icjd→iajb” subscript or, using consecutive letters of the alphabet, the “abcd,ecfd→eafb” subscript, respectively. For reasons of generality, we will employ the latter notation throughout this work, that is, subscripts in alphabetic order “abcd,ecfd→eafb”. We should note that, in our notation convention, the first four indices of the numpy.einsum subscript are reserved for the ERI. Furthermore, these ERI indices need to be expanded in case of Cholesky-decomposed ERI going from “abcd” to “xac,xbd”.

## 3 Memory Management

As system sizes and/or atomic basis sets increase, the GPU memory becomes a limiting factor. In contrast to the CPU side, storing the full multidimensional array on the VRAM is impossible with current hardware. As a remedy, large tensors are split into smaller pieces, then these fragments are moved to the GPU side, where the tensor contraction operations are performed, followed by clearing the GPU memory and then moving the temporary results to the CPU side (see also Figure 1 for a schematic representation). Such batching protocols allow us to handle larger system sizes when the available amount of CUDA memory is smaller than the formally required amount. In the following, we will scrutinize two different batching protocols. First, we discuss our optimized batching recipe, which further accelerates the bottleneck operation of CCD/CCSD calculations compared to our previous work.^25^ Then, we will introduce a generic batching scheme for arbitrary tensor contractions that are encountered in CCD/CCSD calculations and that support both dense and Cholesky-decomposed ERI, yielding a Pythonic CC implementation that almost exclusively runs on the GPU.

**Figure 1:**
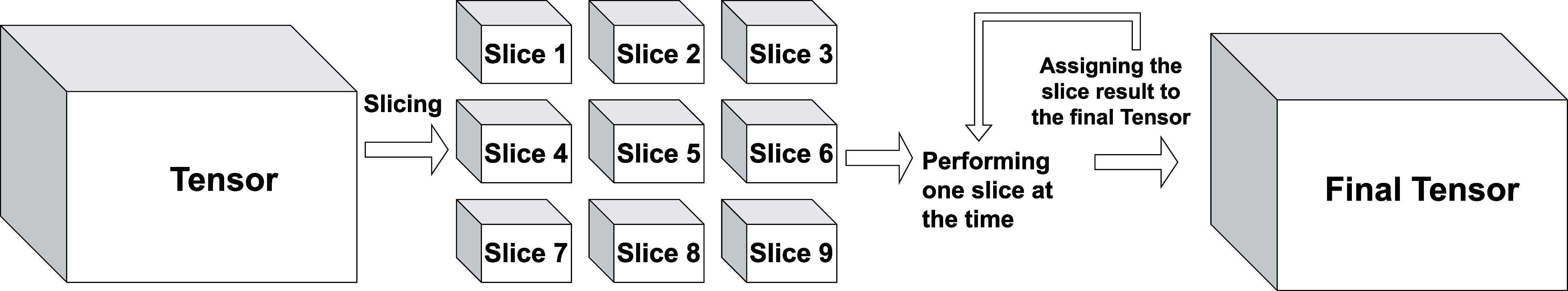
Schematic batching procedure required to process large tensors when going from the CPU to the GPU and back.

### 3.1 An Asymmetric and Dynamic Splitting Protocol

To perform tensor contraction operations involving “gigantic” arrays using the GPU, we have to copy all data involved in the contraction scheme to the VRAM in chunks. In our previous implementation,^25^ we exclusively focused on the CCD/CCSD bottleneck contraction discussed above. We should stress again that this contraction is generally labeled as “abcd,ecfd→eafb”, where the first four letters indicate the Cholesky-decomposed ERI, which can be further translated into “xac,xbd”. Initially, we split the axes “x”, “a”, “b” (input arrays), and “e” (output arrays) into at most three chunks each, with “a” and “b” split homogeneously (same number of chunks).

A schematic representation, depicting the decision-making process of the number of chunks along each axis in question, is shown in Figure 2.

**Figure 2:**
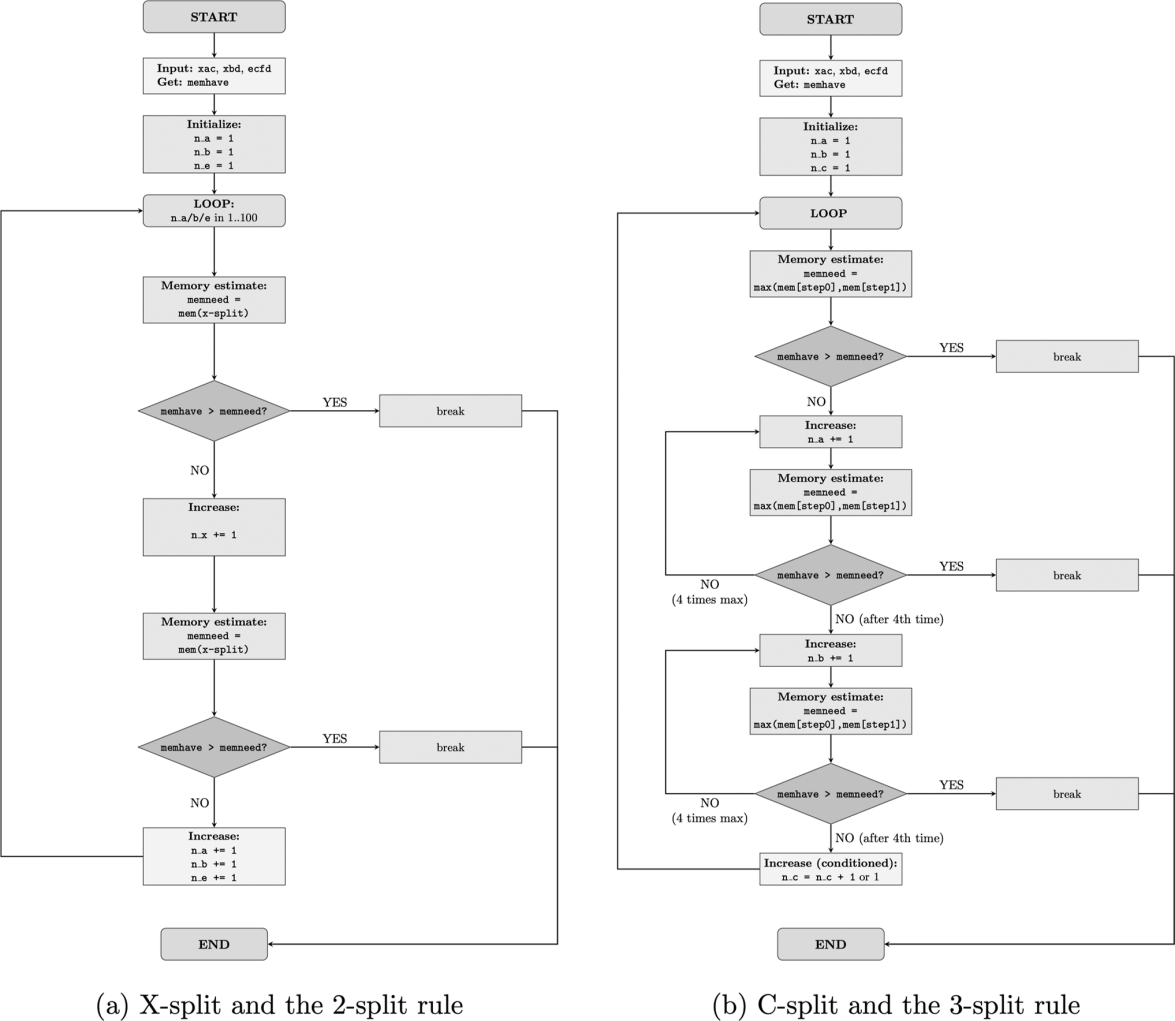
Schematic illustration of the (a) X-split and (b) C-split protocols. “n_x”, “n_a”, “n_b”, “n_c”, and “n_e” indicate the number of batches along the axes “x”, “a”, “b”, “c”, and “e”, respectively. For (a), the number of batches is determined along the “x”, “a”, “b”, and “e” axes. Note that (“a”,“b”,“e”) are split simultaneously according to a 2-split rule (first “x”, then “a”, “b”, and “e”). Mem­(x-split) is the memory compute function that determines the VRAM required for the selected tensor contraction operation. For (b), the number of batches is determined along the “a”, “b”, and “c” axes. “n_a”, “n_b”, and “n_c” indicate the number of batches along the axes “a”, “b” and “c”, respectively. All axes are split asymmetrically according to a 3-split rule (first “a”, then “b”, then “c”). Mem­[step0] and mem­[step1] are the memory compute functions that determine the VRAM required for the selected tensor contraction operation. Note that the split along “c” is conditioned and performed only if the size of the ecfd tensor exceeds 40% of the free and available VRAM (see main text), otherwise the loop is continued without splitting along “c”. All memory functions are defined in Table 1.

Our original implementation was optimized for a specific hardware (NVIDIA V100S), where we screened possible choices in batching for an optimal performance gain.^25^ However, as hardware improves, we need to revisit our original partitioning scheme and carefully validate alternative batching recipes. Current limiting factors are the restriction of the “a” and “b” axes to be indistinguishable, resulting them to be split into the same number of chunks, and treating the summation over “x” (first step, “xac,xbd→acbd”) and the summation over “c” and “d” (second step, “acbd,ecfd→efab”) equivalently. The latter point is particularly crucial as the summations are performed consecutively on the VRAM. The original logic of deducing batch sizes and performing tensor contractions on them was built as three nested for-loops, which yields cubic scaling in the worst-case scenario (in addition to the formal scaling of the tensor contraction operation in question). In our new approach, we generalize the batching and consider the consecutive tensor contractions separately when calculating the required memory on the GPU side. Specifically, we assess whether the tensors in question (as a total or their chunks) would fit on the GPU after separating them into the two steps that are calculated sequentially, namely, (i) the first part consisting of “xac,xbd→acbd”, and (ii) the second part being “acbd,ecfd→efab”. This allows us to adjust the batches more elastically with respect to the size of each tensor. We also transition from splitting the “e” axis to “c”. To improve performance for small and medium-sized tensors, we restrict batching of the axes “a” and “b” as long as possible, splitting the “c” axis only when the size of the ecfd tensor is bigger than 40% of the free and available VRAM on the GPU. Figure 2b summarizes a schematic representation of the optimized asymmetric and dynamic batching protocol, while Table 1 collects the selection rules that determine the required VRAM. We should stress that switching the last batching axis from “e” to “c” was motivated by initial test calculations on the Grace Hopper superchip. However, we do not present separate benchmark calculations for both choices. Instead, the “e” axis is split in the original batching recipe, labeled as “X-split”, while the optimized batching protocol splits the “c” axis, which is indicated as “C-split”. Finally, we should stress that the C-split protocol generates tensor chunks asymmetrically along the split axes. To prevent one tensor from being split into too many small pieces, our protocol increases the number of chunks by at most 4 units before moving on to the next axis. Thus, the proposed C-split works within a 3-split rule, while the original X-split features only 2 splitting conditions. This updated batching protocol contains three very similar, nested for-loops that allow us to batch tensors depending on their unique size. The pseudocode snippet S1 of the Supporting Information summarizes the procedure of splitting all tensor axes, namely, “a”, “b” and “c”. Table 2 summarizes the main algorithmic differences between the X- and C-split algorithms.

**Table 1:** Memory Selection Rules to Assess the Required VRAM Compared to the Available VRAM for the Asymmetric and Dynamic Batching Protocol and Its Generic Extension^a^

splitting	free VRAM	required VRAM	memory function
X-split	avail. VRAM	mem[op0]/(n_chol *n_chol)	
		+ mem[op1]/n_dense	mem(x-split)
		+ mem[out]/(n_chol *n_chol *n_dense)	
C-split	avail. VRAM * 0.98 * 0.9	max(	max(
		mem[xac]/(n_a *n_c)	
		+ mem[xbd]/n_b	mem[step0]
		+2 * mem[op0]/(n_a *n_b *n_c)	
		mem[op0]/(n_a *n_b *n_c)	
		+2 * mem[op1]/n_c	mem[step1])
		+2 * mem[out]/(n_a *n_b))	
		mem[op0]/n	
generic	avail. VRAM * 0.9	+ mem[op1]/n	mem(generic)
		+2 * mem[out]/n	

a As an example contraction, we choose the “abcd,ecfd->efab” (we further denote this expression as op0,op1->out) tensor contraction for X-split and C-split, where the first “abcd” tensor corresponds to Cholesky-decomposed ERIs and has to be expanded to the “xac,xbd” notation. For the X-split algorithm, n_chol is the number of times the op0 tensor is split. Each of the Cholesky-decomposed ERI vectors is divided the same number of times. The amount of op1 batches is determined by n_dense. In the C-split batching protocol, the number of splits of the Cholesky-decomposed ERIs is performed with the help of “n_a”, “n_b” and “n_c”, which correspond to the number of splits along the “a”, “b” and “c” axes, respectively. “n_c” also determines the amount of op1 batches. For the generic algorithm, the tensor contraction is sequenced in optimal pair contractions, where only the first contraction step is batched according to the notation op0,op1->out. mem­[op]: memory in bytes required for operator op. n: number of batches for each operator. The additional prefactor of 2 accounts for intermediates produced by the underlying tensordot operation, as implemented in the GPU library. Note that for the generic case, op0 and op1 are only batched if the underlying tensors contain indices that are not summed over and thus appear in the output indices. Since both tensors are, in theory, batched equally, no distinction into n_0 and n_1 has been made. Free VRAM denotes the VRAM reserved for the contraction operations. Note that for C-split and the generic protocol, the available VRAM (avail. VRAM) is scaled to reserve space for CuPy/PyTorch processes.

**Table 2:** Summary of Axes Involved in the Splitting Process for the Primary Bottleneck Contractions in the CCSD Algorithm, abcd,ecfd->efab, Which Translates to a Two-Step Procedure for Cholesky-Decomposed ERI, Namely, “xac,xbd->acbd” and “acbd,ecfd->efab”

splitting axis	X-split	C-split
x	yes	no
a	yes: simultaneously with b	yes
b	yes: simultaneously with a	yes
c	no	yes
e	yes	no

### 3.2 A Generic Batching Recipe

Based on our previous work and the improved batching recipe discussed above, we devised a generic batching protocol to offload to the GPU any tensor contractions containing both dense and Cholesky-decomposed arrays. We should stress that implementing such a generic batching protocol optimally is notoriously difficult and may be best tackled using machine-learning techniques. In this work, however, we aim for a first, generalizable yet straightforward batching scheme that will even out the required computing times for the bottleneck contraction discussed above and the remaining ones. In our original CPU-GPU hybrid algorithm,^25^ the bottleneck operation in CCD/CCSD calculations was reduced from around 70% to 15% of the total time per CCD/CCSD iteration step. Thus, the bottleneck of the CPU-GPU hybrid implementation shifted to the CPU side that features several “slow”, but “cheaper” (in terms of formal scaling) contractions.

For our generic batching protocol, we assume the following: (i) any tensor contraction involving more than two arrays (for instance, those containing Cholesky-decomposed ERI) are translated into a sequence of two-array contractions. In the following, we will use the general notation op0,op1→out to label one step in the sequence of contractions. (ii) An optimal contraction path, that is, an optimal sequence of two-array contractions at a time, is chosen using numpy.einsum_path. Note that numpy.einsum_path yields a contraction sequence of the lowest cost taking into account the creation of intermediate tensors. If no optimal path is found, an educated guess is made (taking the array sequence as provided in the contraction subscript). (iii) In the batching protocol, we focus exclusively on the first step of the optimal contraction path. For this first contraction step, any tensor contraction of the form a...,b...→a...b... Will be batched along those axes that are not summed over (here, a and/or b) and simultaneously appear in the final output tensor. If no axes are found, we default to the 0-th axis (dense arrays) or the 1-st axis (Cholesky-decomposed ERI; to avoid splits in x). The number of batches is increased symmetrically for both operands (labeled as op0 and op1) until all intermediates fit on the VRAM (see Table 1 for the memory evaluation function). We should note that all subsequent tensor contraction steps are also performed over the batched (intermediate) arrays, until the final step is performed, where the batched result array is added to the correct view of the final result tensor. The decision to batch the first step of the contraction sequence was made deliberatly, as it represents the first optimal step along the complete contraction pathway. The second decision to batch the axes that are not summed over is motivated by our updated asymmetric and dynamic splitting protocol (that is, we avoid splitting the x axis). For reasons of simplicity, we do not support a generic asymmetric batching algorithm yet. A flowchart of the batching protocol is depicted in Figure 3.

**Figure 3:**
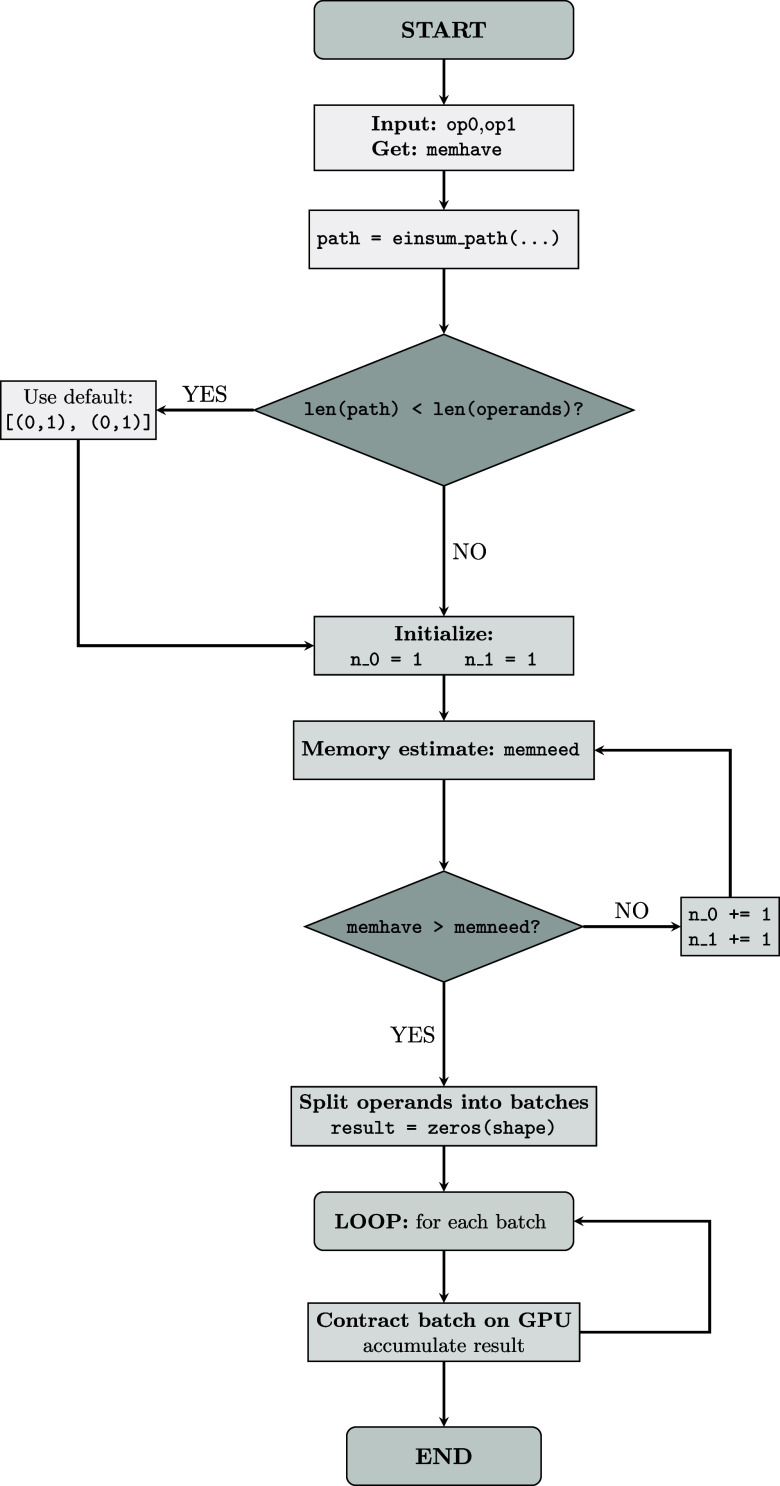
Schematic illustration of the generic batching recipe. The tensor contraction is sequenced in optimal pair contractions (the “find optimal path” decision process), where only the first contraction step is batched according to the notation op0,op1→out. “n_0” and “n_1” indicate the number of batches along the automatically selected axes of “op0” and “op1”, respectively. These axes are not summed over and appear both in the input and output arrays, thus ensuring that the batching procedure is passed on to subsequent steps of the optimal contraction path. memneed is determined through the mem­(generic) memory compute function, which determines the VRAM required for the selected tensor contraction operation, while memhave denotes the accessible VRAM for the current tensor contraction operation. All memory functions are defined in Table 1.

Finally, we should note that we offload tensor contraction operations that contain at least one three-dimensional array. Thus, tensor contractions containing a ${\mathrm{T̂}}_{1}$ vertex are not performed on the GPU if they are to be contracted with another dense array. On the other hand, all tensor contraction operations containing Cholesky-decomposed ERI are offloaded to the GPU, irrespective of the dimensionality of the second operand. Our choice was motivated by molecular benchmark calculations, which indicated that no significant speed-up has been gained compared to the original CPU implementation. However, a detailed analysis of such tensor contractions will be the focus of follow-up work. The pseudocode snippet S2 of the SI summarizes the logical steps of the generic batching protocol.

### 3.3 Optimized CUDA Memory Allocation in CuPy and PyTorch

Allocating and deallocating memory using CUDA APIs is time-consuming. Thus, exploiting CUDA operations like “cudaMalloc” or “cudaFree” with our batching procedures can be costly, especially because the number of batches or slices determines how many memory accesses are required. To avoid an unnecessary number of CUDA driver calls, PyTorch and CuPy utilize caching memory allocators for tensors. These allocators optimize memory allocation: whenever a tensor is deallocated, the allocated memory is not returned to the GPU. Instead, this block of memory is reserved in the cache. Then, when we need to allocate another tensor, this cache is checked first, and the caching memory allocator ensures that enough memory is available. This optimized memory allocation procedure speeds up calculations on the GPU side, but might not be as efficient as calling “cudaMallocAsync” or “cudaFreeAsync” directly.

### 3.4 A Generic Interface for a Dynamic Switching between CuPy, PyTorch, and CPU-Only Libraries

Since all CC calculations are dominated by tensor contraction operations, it is desirable to design CC implementations that can exploit any tensor contraction library without changing the codebase. Based on this idea, PyBEST allows us to define complex array operations concisely using the Einstein summation convention without confining the linear algebra operations to specific library choices. This allows for a direct comparison of tensor contraction libraries for the same CC codebase.

Considering GPU-acceleration, we always exploit the same batching strategies (discussed above) to optimize the memory handling of tensor contractions on the GPU side. For ease of use and an optimal flow of logic, we designed and implemented a general GPU-ready tensor contraction engine to change the GPU acceleration background on the fly. In this work, we will focus on two libraries that provide a Python frontend or Python-based API, namely CuPy and PyTorch. However, our flexible GPU-ready tensor contraction engine can be easily interfaced with other libraries for GPU-accelerated computing as well (for example, TensorFlow^28^). Currently, our generic tensor contraction function supports tensor operations with a NumPy,^31^ CuPy,^29^ PyTorch,^30^ or C++ backend.

As a least invasive solution, environment variables determine the library choice for GPU acceleration. Figure 4 shows a flowchart of the implemented GPU-supported tensor contraction logic. First, we check if a CUDA-ready GPU is available and test basic CUDA operations (allocation and deallocation). If unavailable or an assertion is raised, we switch to CPU-only tensor contraction libraries. If implemented and/or supported, numpy.tensordot is taken as the default contraction engine. Otherwise, opt_einsum or, as a last resort, numpy.einsum is selected (see also ref 25 for more details). At the GPU side, we opt for the CuPy- or PyTorch-based tensordot implementations, while the final output tensors are brought into proper shape using the transpose (CuPy) or permute (PyTorch) functions. Once the library for GPU acceleration is chosen (CuPy or PyTorch), a batching recipe is selected. Currently, the three choices described above are supported and cover the “C-split” or “X-split” logic for selected bottleneck contractions or the generic batching protocol. This flexible approach yields a modular tensor contraction engine that facilitates testing different libraries for GPU acceleration with minimal changes to the codebase.

**Figure 4:**
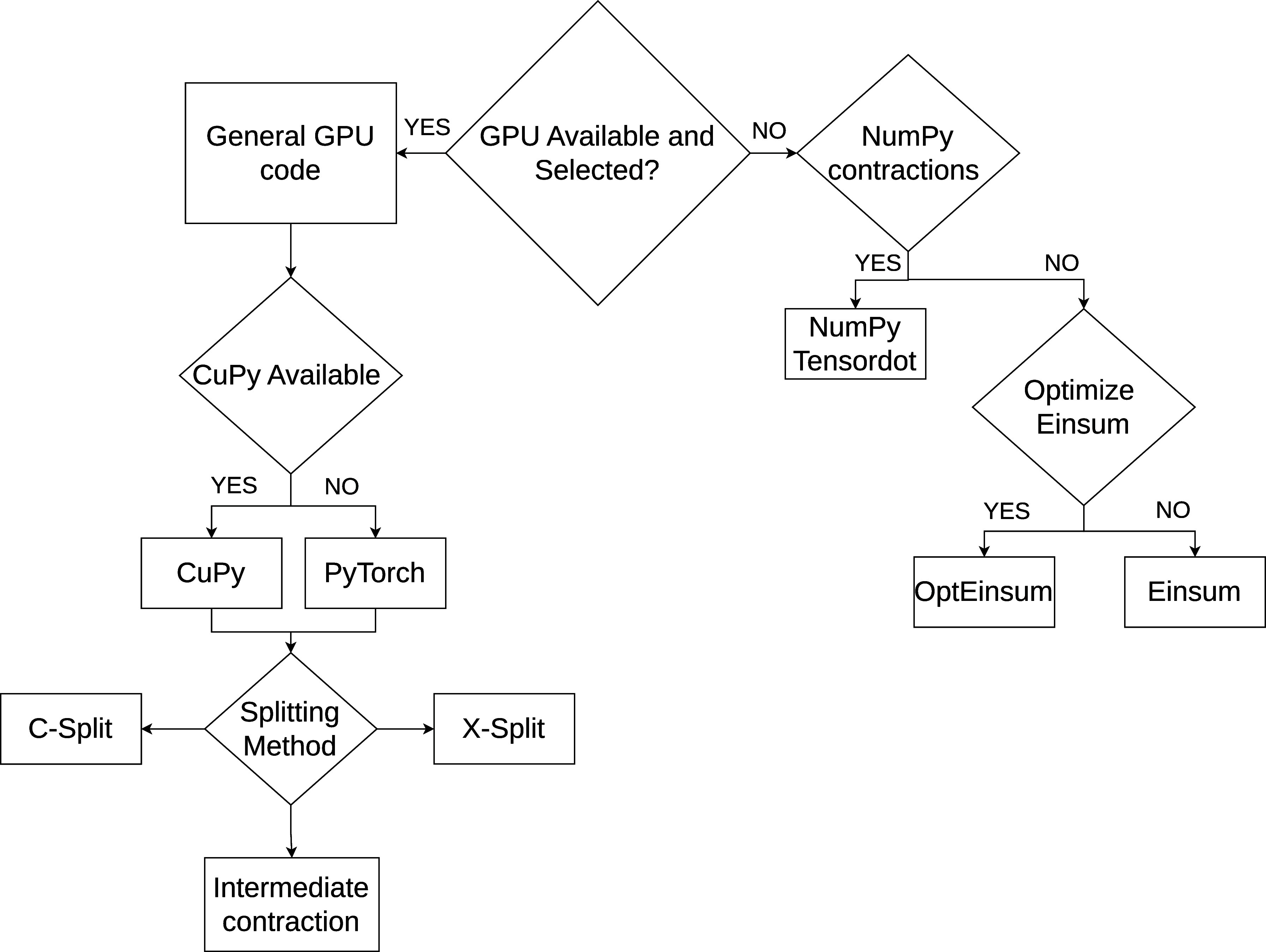
Schematic representation of the logic flow of the designed tensor contraction engine in PyBEST.

## 4 Computational Details

All the contraction benchmarks and quantum chemical calculations have been carried out in a developer version of the PyBEST^36,37^ software package (v2.2.0.dev0). These computations were performed on a single NVIDIA H100 and GH200 architectures available on the LEM computing cluster at the Wrocław Centre for Networking and Supercomputing^52^ (WCSS) and the Helios supercomputer cluster at the Academic Computer Centre Cyfronet AGH,^53^ respectively. These computing facilities are part of the Polish Grid Infrastructure (PLGrid). A detailed description of each GPU/CPU node’s hardware and software specifications is provided in Table 3.

**Table 3:** Comparison of NVIDIA’s GH200 (Helios@Cyfronet) and H100 (Lem@WCSS) Hardware and Software Specifications

parameter	GH200	H100
CPU RAM [GB]	480	1006
CUDA Cores	16896	16896
total VRAM [MiB]	97871	95830
max graphics clock [MHz]	1980	1980
max memory clock [MHz]	2619	1593
power limit (TDP) [W]	900	700
CUDA driver version	575.57.08	570.195.03
CUDA toolkit	12.6.20	12.4.99
PCIe generation (current)	4	5
PCIe generation (max)	4	5
PCIe width (current)	1	16
PCIe width (max)	1	16
python version	3.11.5	3.11.3
NumPy version	2.1.3	2.3.3
CuPy version	13.3.0	13.6.0
PyTorch version	2.5.1+cu124.post2	2.8.0+cu128

In the contraction benchmark calculations, we tested randomly generated numbers, with timings averaged over five individual runs. The first iteration is usually slower because the library must initialize the CUDA driver and its related components. To avoid this, we run the GPU jobs with small data allocations first, prior to the main calculations.

In all molecular calculations, we used the Cholesky-decomposed two-electron integrals with a predefined threshold (10^–5^ unless stated otherwise), and the core orbitals (1s for C, N, and O) were kept frozen. For the a decameric water cluster— ${{\left(H\right)}_{2}O)}_{10}$ (**1**) we used the cc-pVDZ and cc-pVTZ basis sets.^54^ The 6–31+G** basis set^55–57^ was used for the a hydrated methylated uracil dimer (mU)_2_H_2_O complex (**2**) to compare with existing reference GPU benchmarks. For the donor–π–acceptor dye 4-(diphenylamino)­phenylcyanoacrylic acidmolecule—L0 dye (**3**) we employed the cc-pVDZ, cc-pVTZ,^54^ and aug-cc-pVDZ basis sets.^58^ The xyz structure for all investigated molecules are visualized with PyBEST GUI^59^ in Figure 5.

**Figure 5:**
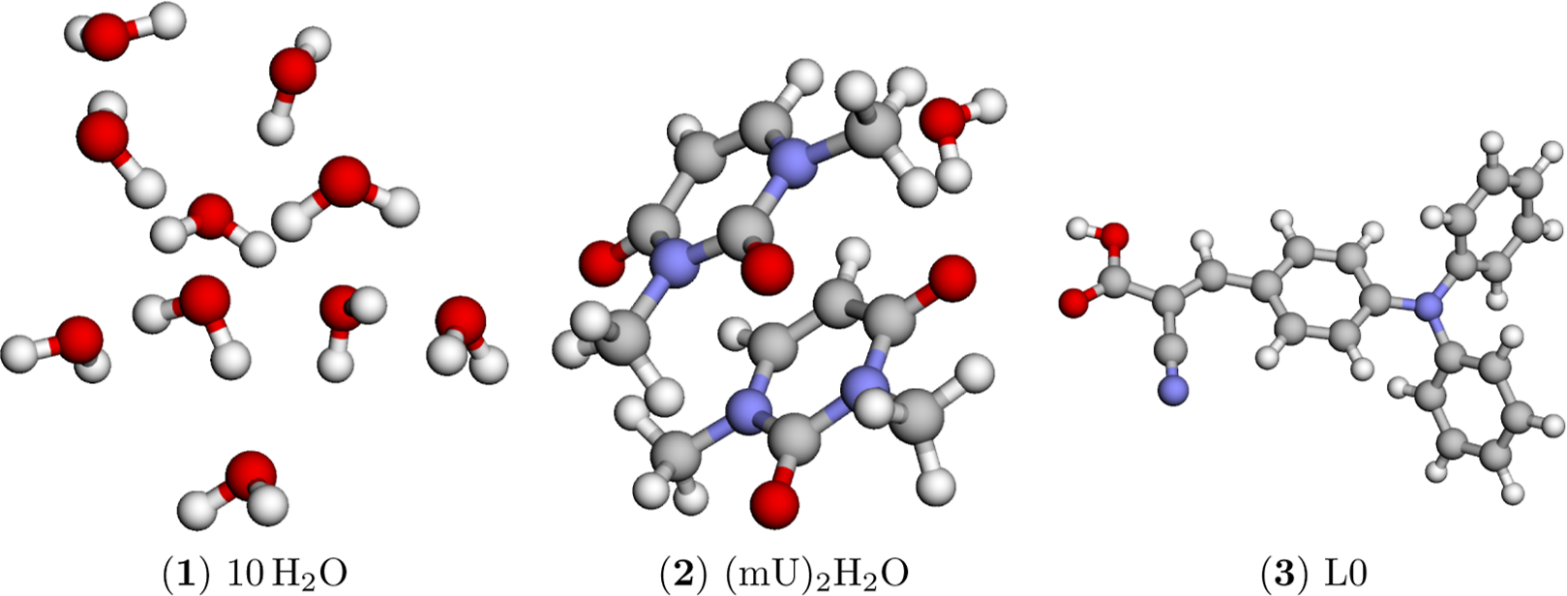
Structures of the investigated molecules drawn with the PyBEST GUI. Atom types are color-coded: white for hydrogen, red for oxygen, gray for carbon, and purple for nitrogen.

## 5 Results and Discussion

### 5.1 Contraction Benchmarks with Predefined Dimensions and Randomly Generated Arrays

First, we examine GPU performance using controlled synthetic benchmarks with predefined tensor dimensions and randomly generated data (cf. Table 4 for details) before proceeding to realistic molecular test cases.^60,61^ Specifically, we assess the performance of the tensor contraction abcd,ecfd→efab on GPUs across a range of basis set sizes (800–1300 basis functions) and numbers of occupied orbitals (100 and 150). The benchmarks were carried out using both PyTorch- and CuPy-based implementations of the X-split and C-split algorithms (see Section 3 for details on the memory-efficient splitting strategies). To mimic the computationally dominant contraction step in CCSD for medium-to-large molecules, the number of Cholesky vectors was fixed at 5·*N*_basis_, corresponding to an approximate decomposition threshold of 10^–5^. Performance results obtained on the NVIDIA GH200 and H100 GPU architectures are shown in the upper and lower panels of Figure 6, respectively. The numerical data underlying this figure are provided in Table 4.

**Figure 6:**
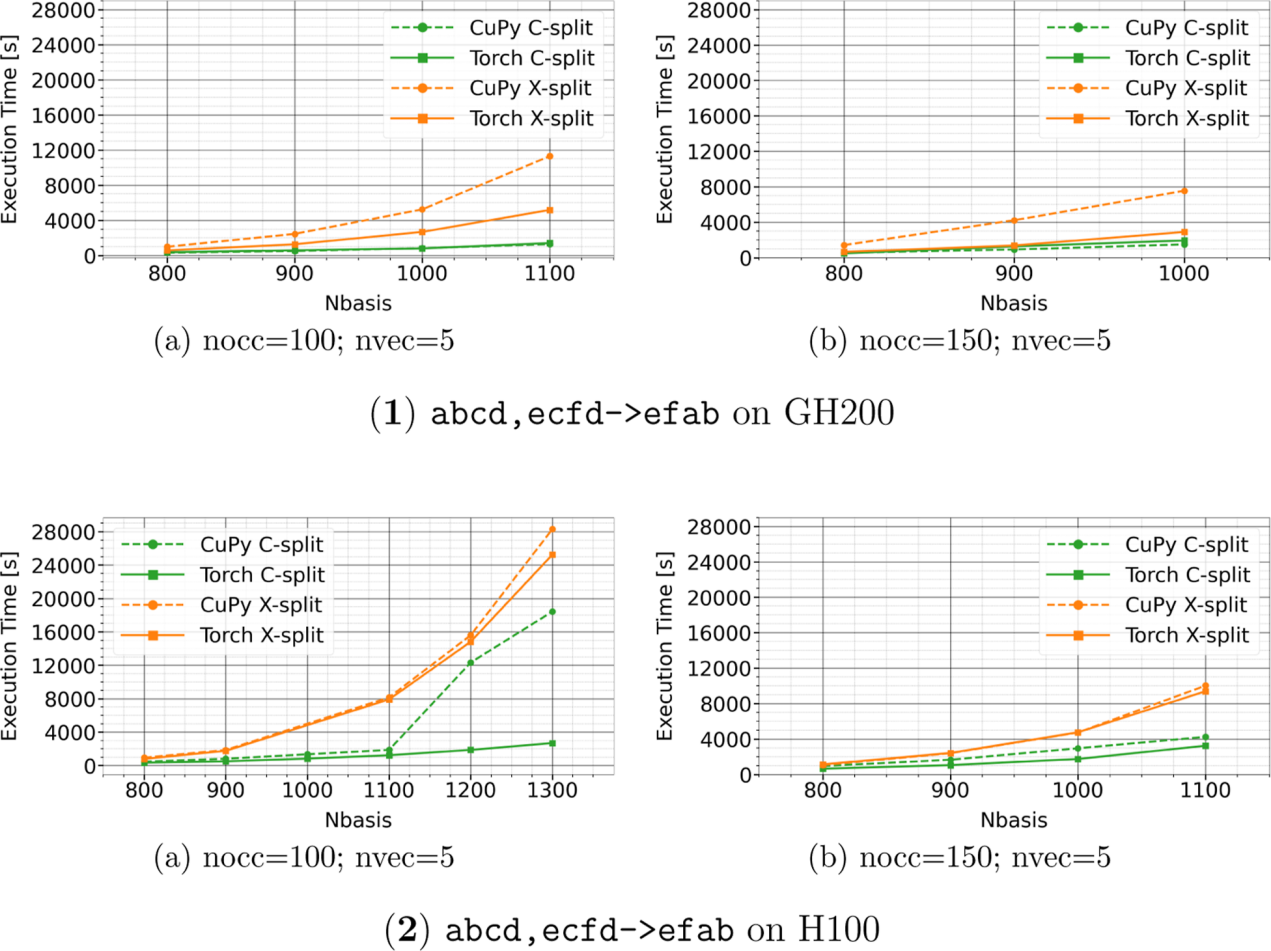
Comparison of CuPy- and PyTorch-based C- and X-splits execution times for the abcd,ecfd→efab contraction (vvvv block) on GH200 and H100 GPU architectures. The number of occupied orbitals (nocc) and the number of Cholesky vectors (nvec) in units of *N*_basis_ is denoted explicitly below each graph.

**Table 4:** Comparison of X-Split and C-Split Performance on H100 and GH200 GPU Architectures for the abcd,ecfd->efab Bottleneck Contraction for the Particle–Particle Ladder Term Using Predefined Dimensions and Randomly Generated Arrays with CuPy and PyTorch^a^

					GH200 (avg of 5)		H100 (avg of 5)	
**N**	**nocc**	**nvec**	**nvir**	**library**	**X-split [s]**	**C-split [s]**	**X-split [s]**	**C-split [s]**
800	200	10 × N	600	PyTorch	834.7	1573.6	1568.1	1485.3
				CuPy	2061.3	706.8	1068.4	1364.7
900	225	10 × N	675	PyTorch	n.c	n.c	3628.4	3042.2
				CuPy	n.c	n.c	13725.0	2529.6
1000	250	10 × N	750	PyTorch	n.c	n.c	n.c	6233.1
				CuPy	n.c	n.c	n.c	7759.1
800	100	5 × N	700	PyTorch	574.7	355.9	789.4	330.5
				CuPy	987.7	306.6	933.4	461.5
900	100	5 × N	800	PyTorch	1265.7	587.1	1753.3	496.2
				CuPy	2450.2	495.0	1853.0	809.7
1000	100	5 × N	900	PyTorch	2673.7	805.8	n.c	827.1
				CuPy	5241.7	829.1	n.c	1338.7
1100	100	5 × N	1000	PyTorch	5177.4	1401.7	7907.7	1223.4
				CuPy	11305.0	1282.7	8151.5	1860.9
1200	100	5 × N	1100	PyTorch	n.c	n.c	14821.7	1864.7
				CuPy	n.c	n.c	15601.2	3042.4
1300	100	5 × N	1200	PyTorch	n.c	n.c	25244.9	2679.1
				CuPy	n.c	n.c	28292.9	4665.3
800	150	5 × N	650	PyTorch	637.4	475.3	1128.1	651.2
				CuPy	1410.7	532.8	1050.2	940.9
900	150	5 × N	750	PyTorch	1364.7	1261.1	2419.8	1044.4
				CuPy	4213.1	911.6	2390.6	1654.1
1000	150	5 × N	850	PyTorch	2886.4	1924.4	4749.6	1739.7
				CuPy	7536.1	1489.2	4727.1	2945.2
1100	150	5 × N	950	PyTorch	n.c	n.c	9384.7	3234.4
				CuPy	n.c	n.c	10024.0	4241.5
1000	50	10 × N	950	PyTorch	5262.6	574.6	n.c	673.0
				CuPy	n.c	763.0	n.c	1006.4
1200	50	10 × N	1150	PyTorch	n.c	1756.0	n.c	2031.5
				CuPy	n.c	2155.2	n.c	2429.5

a nocc: number of occupied orbitals. nvir: number of virtual orbitals. nvec: number of Cholesky vectors. N: number of basis functions (nocc+nvir). n.c.: not computed due to insufficient memory on the CPU side or OutOfMemoryError for X-split. We should note that the memory function for X-split in Table 1 was optimized for medium-sized problems. For reasons of reproducibility and a direct comparison with our original work,^25^ we did not re-optimize it in this work. Note that all GPU times include data preparation (batching on the CPU side), data transfer, and the actual algebraic operations

In all cases examined in Figure 6, the X-split algorithm—originally optimized for the NVIDIA V100S with 32 GB VRAM—is substantially slower than the newly introduced C-split algorithm. This holds true across both CuPy and PyTorch implementations and for both the GH200 and H100 GPU architectures. Both CuPy and PyTorch exhibit markedly better performance on the GH200 compared to the H100, as clearly visible when comparing the upper and lower panels of Figure 6. Notably, on the GH200, the combination of CuPy with X-split remains computationally more efficient than PyTorch with X-split, whereas the two libraries perform very similarly when using X-split on the H100. The performance advantage of C-split over X-split becomes more pronounced as the number of (virtual) basis functions increases (corresponding, in our test suite, to fewer occupied orbitals). On the GH200 (upper panel of Figure 6), the difference in performance between CuPy-based and PyTorch-based C-split implementations is negligible. In contrast, this difference is significantly larger on the H100, particularly for the larger *N*_basis_ dimension shown in the lower-left part of the figure. In these cases, the combination of PyTorch with C-split substantially outperforms that of CuPy and C-split, yielding considerably shorter execution times. In the largest case with *N*_basis_ = 1300, the C-split performs 32, 7, 9 slices (corresponding to n_a, n_b, and n_c on Figure 2b) and X-split executes 20, 19 slices (corresponding to n_a = n_b, and n_e on Figure 2a), respectively.

In summary, a significant speed-up in execution times for the abcd,ecfd→efab contraction is obtained by combining PyTorch with the new C-split algorithm. The PyTorch-based C-split gives speed-ups of up to a factor of 10 compared to our initial CuPy-based X-split implementation.^25^ Furthermore, the use of the NVIDIA GH200 and H100 GPU architectures overcomes the previous limitation on the maximum number of basis functions that can be handled on a single GPU, compared to earlier hardware generations such as the NVIDIA V100S (32 GB).^25^ Altogether, this strongly motivates further exploration and application of this approach to realistic molecular test sets.

### 5.2 Molecular Benchmark Calculations

Our molecular test set comprises three systems: a decameric water cluster ${{(H}_{2}O)}_{10}$ labeled as (**1**), a hydrated methylated uracil dimer, (mU)_2_·H_2_O molecule labeled as (**2**), and the donor–π–acceptor dye 4-(diphenylamino)­phenylcyanoacrylic acid abbreviated as L0 and labeled as (**3**) in different basis sets (cf. Section 4). Corresponding molecular structures are presented in Figure 5. These systems are established benchmarks with published reference CCSD timing results from prior CPU and GPU implementations.

The performance of our newly developed CC implementation within PyBEST and C-split is reported and compared with existing references from Psi4 and TeraChem in Table 5. Specifically, we analyze the timing of a single CCSD iteration step and the average time spent on GPU function calls. Note that not all CCSD calculations are performed exclusively on the GPU unless explicitly stated. Furthermore, the GPU-accelerated CCSD calculations within PyBEST combine the C-split and generic batching protocols and simultaneously exclude all tensor contractions that cannot be performed using tensordot or contain a dense ERI tensor contracted with a ${\mathrm{T̂}}_{1}$ vertex. All these special cases are still restricted to the CPU.

**Table 5:** Average Timings [S] of a CCSD Iteration Step Compared to Other GPU Computations and Average GPU Time Used in one Iteration for the Investigated Molecules (**1**), (**2**), and (**3**) and Different Basis set Sizes^a^

basis set	software	refs	CPU cores	GPU	CCSD	avg. GPU
(H_2_O)_10_, 30 atoms						
**cc-pVDZ 240 AOs**	Psi4/DF	60	16	-	16s	-
	PyBEST/CD	25	36	-	337s	-
	PyBEST/CD/CuPy	25	36	tesla V100S	92s	4.4s
	PyBEST/CD/Grace	this work	72	-	52s	-
	PyBEST/CD/PyTorch	this work	1	H100	31.7s	21s
	PyBEST/CD/CuPy	this work	1	H100	48s	37s
	PyBEST/CD/PyTorch	this work	72	GH200	25.4s	17s
	PyBEST/CD/CuPy	this work	72	GH200	23.9s	15.8s
	TeraChem/CD	61	1	tesla V100	10s	10s
**cc-pVTZ 580 AOs**	PyBEST/CD/PyTorch	this work	1	H100	6.4m	4.7m
	PyBEST/CD/CuPy	this work	1	H100	9.6m	7.9m
	PyBEST/CD/PyTorch	this work	72	GH200	5.7m	4.6m
	PyBEST/CD/CuPy	this work	72	GH200	5.5m	4.3m
(mU)H_2_H_2_O, 39 atoms						
6–31+G** 468 AOs	PyBEST/CD	25	36	-	96.5m	-
	PyBEST/CD/CuPy	25	36	tesla V100S	33.2m	4.3m
	PyBEST/CD/Grace	this work	72	-	18.2m	-
	PyBEST/CD/CuPy	this work	1	H100	9.3m	6.8m
	PyBEST/CD/PyTorch	this work	1	H100	6.8m	4.3m
	PyBEST/CD/CuPy	this work	72	GH200	6.1m	4.5m
	PyBEST/CD/PyTorch	this work	72	GH200	6.7m	5m
	TeraChem/CD	61	1	Tesla V100	2.5m	2.5m
L0, 42 atoms						
**cc-pVDZ 444 AOs**	PyBEST/CD	25	36	–	45.9m	–
	PyBEST/CD/CuPy	25	36	tesla V100S	15.3m	2.0m
	PyBEST/CD/Grace	this work	72	-	8.5m	-
	PyBEST/CD/PyTorch	this work	1	H100	3.2m	2.1m
	PyBEST/CD/CuPy	this work	1	H100	4.5m	3.3m
	PyBEST/CD/PyTorch	this work	72	GH200	3.3m	2.5m
	PyBEST/CD/CuPy	this work	72	GH200	3.1m	2.4m
**aug-cc-pVDZ 742 AOs**	PyBEST/CD/PyTorch	this work	1	H100	19.5m	15.1m
	PyBEST/CD/CuPy	this work	1	H100	31.2m	26.9m
	PyBEST/CD/PyTorch	this work	72	GH200	17.1m	12.8m
	PyBEST/CD/CuPy	this work	72	GH200	19.7m	15.2m
**cc-pVTZ 1004 AOs**	PyBEST/CD/PyTorch	this work	1	H100	n.c	n.c
	PyBEST/CD/CuPy	this work	1	H100	139.7m	54.6m
	PyBEST/CD/CuPy	this work	32	H100	63.7m	50.8m
	PyBEST/CD/PyTorch	this work	72	GH200	n.c	n.c
	PyBEST/CD/CuPy	this work	72	GH200	52.8m	33.6m
	PyBEST/CD/CuPy/GPU-only	this work	72	GH200	59.6m	40.0m

a One iteration step contains the evaluation of the vector function, the update of the CC amplitudes, and the evaluation of the CC energy expression. All timings correspond to differences in epoch times. All PyBEST results are shown for the CPU-only (if available) and various CPU-GPU hybrid variants. The Psi4 data is given as a comparison. CCSD: mean value for the time of one CC iteration averaged over 4 steps. avg. GPU: mean value for the time spent in the GPU function call, averaged over 4 steps CD: Cholesky decomposition. DF: Density Fitting. PyBEST/CD/Grace: Average wall-clock time (over 5 steps) for a CPU-only run on the GH200 superchip. For the largest system, we encountered internal errors with PyTorch; these cases are marked as “n.c.” (not converged). Note that all GPU times include data preparation (batching on the CPU side), data transfer, and the actual algebraic operations

For the water cluster (**1**) with the cc-pVDZ basis set, we achieved a significant improvement in CCSD performance on the GH200 superchip, reaching approximately a 6-fold speedup compared to our initial PyBEST CPU implementation (reducing the time per CCSD iteration from 337 to 57 s). The 57 s per iteration obtained with PyBEST/CD/Grace using 72 CPU cores should be compared with the C++ implementation in Psi4 running on 16 CPU cores. For GPU calculations on the same system, we observe an approximately 4-fold speedup with the C-split approach relative to our original CuPy/X-split implementation, reducing the time per CCSD iteration from 92 to 24 s^25^ These results are highly encouraging, considering that the fully optimized bare-CUDA implementation in TeraChem achieves approximately 10 s per CCSD iteration for the same system—our Python-based implementation is only 2.4 slower.

For both the cc-pVDZ and cc-pVTZ basis sets, the lowest average GPU timings are obtained with the CuPy implementation on GH200. In contrast, on the H100 platform, the PyTorch implementation is nearly 50% faster than CuPy. One possible explanation is that, for problem sizes of this scale, PyTorch is more effective at hiding PCIe communication overheads by overlapping data transfers with computation, thereby reducing idle time associated with PCIe synchronization. However, without detailed backend-level profiling and kernel analysis, it is difficult to draw definitive conclusions regarding the exact origin of the observed performance differences.

A similar PyTorch/CuPy performance trend is observed for the uracil dimer monohydrate (**2**) and the L0 molecule (**3**) using the cc-pVDZ basis set. In analogy to system (**1**), for molecule (**2**) the best-performing PyBEST GPU implementation is only approximately 2.4× slower than the highly optimized native CUDA implementation in TeraChem. Interestingly, when the number of basis functions exceeds 700, as in the aug-cc-pVDZ basis set, the PyTorch implementation begins to outperform CuPy in terms of execution time. However, for the largest investigated system (molecule (**3**) with the cc-pVTZ basis set and more than 1000 basis functions), only CuPy computations are feasible. We attribute the observed differences to the internal implementation details of the PyTorch and CuPy backends, which are complex and difficult to fully disentangle without in-depth profiling (for instance, using NVIDIA Nsight). In particular, PyTorch employs a highly sophisticated CUDA caching allocator specifically designed to maximize memory reuse and minimize fragmentation under dynamic allocation patterns. Although CuPy also implements a memory pool, its allocation strategy is considerably simpler and tends to be less efficient in highly dynamic workloads. Additionally, PyTorch benefits from extensively optimized custom kernels and advanced execution strategies, while CuPy relies more directly on standard CUDA and cuBLAS primitives. These fundamental differences may not only affect runtime performance and memory consumption but can also explain why CuPy succeeds in certain large-tensor cases where PyTorch encounters failures.

Importantly, we also see clear evidence that, for this large system, the CPU part of the computations becomes important: CuPy calculations on H100 with 32 CPUs reduce the timing by half compared to a single CPU. That suggests the particle–particle ladder term is no longer the only bottleneck operation.

For the largest basis set in the L0 test case, we also performed computations using a fully generic GPU path, lifting the restriction on contractions involving ${\mathrm{T̂}}_{1}$ vertices (note that only tensordot-ready contractions are offloaded to the GPU). This approach provides similar timings as the restricted GPU variant (excluding all ${\mathrm{T̂}}_{1}$ terms from being offloaded to the GPU), albeit the GPU-only variant performs slightly worse. We should stress here that the GPU-acceleration performance is substantially better on NVIDIA GH200 than H100 for *N*_basis_ ≥ 1000. Furthermore, 30% of the computing time of the GPU-only approach is still performed on the CPU side. These operations include data preparation (about 7 min; expanding the symmetry-unique ${\mathrm{T̂}}_{2}$ amplitudes to their dense representation to allow for NumPy manipulations) and other tensor contractions (around 13 min) that cannot be performed using the tensordot method. Examples for the latter are the creation of the ${\mathrm{t̃}}_{kl}^{cd}=t_{k}^{c}t_{l}^{d}$ intermediate (during the residual evaluation) or the extraction of the pair amplitudes {*t*_*kk*_^*cc*^} from {*t*_*kl*_^*cd*^} (during energy calculation). Our calculations suggest that these operations (data manipulations, intermediate creation) become a significant bottleneck on the CPU side for large numbers of basis functions. Whether they can be efficiently offloaded to the CPU requires, however, further evaluation. Thus, for large enough basis set sizes, we observe another shift in the bottleneck operations, moving from tensor contractions to data preparation. Finally, we should stress that a direct comparison to CPU-only data is difficult primarily because the CPU-only counterpart implementations are computationally too time-consuming and exceed the available computing time on the underlying HPC infrastructure.

## 6 Comparisons with Other Python-Based GPU Implementation Strategies in Quantum Chemistry Codes

Parallel to our work, several groups have actively developed GPU-accelerated Python-based quantum chemistry packages. Two notable efforts are ByteQC^9^ and GPU4PySCF.^8^ ByteQC is a comprehensive library comprising six subpackages focused on accelerating various many-body quantum chemistry methods. Among these, the “cucc” subpackage is specifically dedicated to coupled-cluster theory, supporting both CCSD and CCSD­(T). In recent benchmarks on NVIDIA A100 GPUs (80 GB VRAM), ByteQC achieved speedups of 20–30× over a 100-core CPU implementation for CCSD. The authors further demonstrated calculations on systems containing up to 1610 orbitals in the cc-pVQZ basis set—to the best of our knowledge, the largest GPU-accelerated CCSD calculation reported in the literature to date. In addition, they reported multi-GPU scaling with speedups of approximately 1.5× using two GPUs, gradually increasing toward 2.0× with more GPUs. Although we achieve speed-ups up to a factor of 3 compared to our latest CPU-only implementation, the overall wall times for a GPU-accelerated CCSD iteration step are similar for comparable system sizes (see the various examples for water clusters in ref 9).

GPU4PySCF^8^ aims to accelerate the widely used PySCF quantum chemistry package through GPU computing. It provides GPU support for key functionalities, including DFT, geometry optimization, vibrational frequency analysis, implicit solvent models, and density fitting. However, the latest release (v1.7.0) offers only experimental GPU acceleration for CC methods.

## 7 Conclusions and Outlook

In this work, we introduced new batching algorithms, namely, the asymmetric and dynamic C-split protocol and a fully generic splitting algorithm, to efficiently offload the bottleneck CCSD contraction to the GPU in the PyBEST software package (cf. Figure 2). That, in combination with the NVIDIA H100 and GH200 GPU architectures, allows us to obtain a significant speedup for both the CCSD bottleneck contraction and CCSD vector function evaluation compared to our previous implementations.^25^ Specifically, combining the C-split algorithm with PyTorch yields speedups of up to a factor of 10.

The C-split batching algorithm was applied to a molecular test set consisting of ${{(H}_{2}O)}_{10}$ , (mU)_2_·H_2_O, and L0 (Figure 5). For these systems, we observe a substantial reduction in the CCSD iteration time. For small to medium systems (up to 500 basis functions), CuPy on the NVIDIA GH200 provides the best performance, yielding overall speedups of 4–5 for the CCSD iteration step. For these systems, PyTorch on H100 is consistently faster than CuPy, whereas CuPy performs slightly better on GH200. For L0 in the aug-cc-pVDZ basis set (742 basis functions), PyTorch becomes slightly faster than CuPy on GH200. For the largest system investigated—L0 in the cc-pVTZ basis set (exceeding 1000 basis functions)—only CuPy timings are presently available. Here, the CCSD iteration time on the GH200 is reduced by approximately 60% relative to H100, consistent with the GH200s superior specifications, including higher peak memory bandwidth, larger on-GPU HBM capacity, and the high-bandwidth coherent NVLink-C2C interconnect to unified CPU memory.

Following targeted optimizations of the scaling-determining tensor contraction in the CCSD procedure within PyBEST, secondary operations (e.g., numpy.einsum-restricted operations, residual builds, data preparation) now contribute more significantly to the total runtime. Further performance gains will therefore require optimization of these supporting kernels. These benchmarks demonstrate that the choice between CuPy and PyTorch for maximal CCSD acceleration in PyBEST, specifically, or Python, in general, is not trivial: optimal backend selection depends sensitively on system size, basis set size, contraction pattern, and hardware platform (H100 vs GH200). In general, the optimal choice between the C-split and X-split strategies is highly nontrivial and depends sensitively on the particular contraction scheme, tensor dimensions, memory layout, and hardware characteristics. Accurately predicting the optimal strategy would likely require a dedicated machine-learning-based performance model, which we intend to investigate in future work. This challenge further motivates the continued development and optimization of our GPU interface, including more accurate memory estimation, improved data-transfer scheduling, and optimized array transposition strategies, all of which directly affect the efficiency of the splitting procedure. Consequently, at the current stage of development, we cannot yet recommend a universal batching strategy that consistently performs optimally across all tensor contraction schemes. Nonetheless, our initial data suggest that large tensors benefit from being split asymmetrically. This, however, needs to be confirmed in future studies.

Finally, our molecular calculations suggest that further optimizing the CCSD bottleneck contraction does not necessarily result in an overall performance gain. The numerous “faster” tensor contractions are considerably harder to accelerate by offloading to the GPU. Additional effort needs to be put into further accelerating nonbottleneck contractions and refining the generic batching recipe to maximize performance gain. Besides machine-learning techniques, performance has to be maximized using dedicated profiling tools, such as NVIDIA Nsight or similar. Future development will focus on leveraging multi-GPU parallelism on both the NVIDIA H100 and GH200 architectures, including exploitation of NVLink domain-spanning capabilities on GH200 systems to potentially enable larger-scale coupled-cluster calculations on systems with thousands of basis functions.

## Supplementary Material





## Data Availability

The data underlying this study are available in the published article. The PyBEST code is available on Zenodo at https://zenodo.org/records/10069179 and on PyPI at https://pypi.org/project/pybest/.

## Notes

The following data are available free of charge. The Supporting Information is available free of charge at https://pubs.acs.org/doi/10.1021/acs.jctc.6c00558.

SI.pdf: pseudocode for C-split and the generic batching protocol (PDF)
benchmarks.xlsx: raw data of the presented benchmark calculations (XLSX)

## ⊥

Department of Mathematics and Computer Science, Freie Universität in Berlin, Germany

## References

[1] LeGrand, S. ; Scheinberg, A. ; Tillack, A. F. ; Thavappiragasam, M. ; Vermaas, J. V. ; Agarwal, R. ; Larkin, J. ; Poole, D. ; Santos-Martins, D. ; Solis-Vasquez, L. ; GPUaccelerated drug discovery with docking on the summit supercomputer: Porting, optimization, and application to COVID-19 research. Proceedings of the 11th ACM international conference on bioinformatics, computational biology and health informatics; ACM, 2020, pp 1–10.

[2] Manathunga M., Aktulga H. M., Götz A. W., Merz Jr K. M. (2023). Quantum mechanics/molecular
mechanics simulations on NVIDIA and AMD graphics processing units. J. Chem. Inf. Model..

[3] Yu Y., Cai C., Wang J., Bo Z., Zhu Z., Zheng H. (2023). Uni-Dock:
GPU-accelerated docking enables ultralarge virtual screening. J. Chem. Theory Comput..

[4] Lukač N., Mongus D., Žalik B., Štumberger G., Bizjak M. (2024). Novel GPU-accelerated high-resolution solar potential
estimation in urban areas by using a modified diffuse irradiance model. Appl. Energy.

[5] Hu F., Niezgoda S., Xue T., Cao J. (2025). Efficient GPU-computing
simulation platform JAX-CPFEM for differentiable crystal plasticity
finite element method. npj Comput. Mater..

[6] Wang H., Suyu S. H., Galan A., Halkola A., Cappellari M., Shajib A. J., Cernetic M. (2025). GPU-Accelerated Gravitational
Lensing
and Dynamical (GLaD) modeling for cosmology and galaxies. A & A.

[7] Wu X., Koslowski A., Thiel W. (2012). Semiempirical quantum chemical calculations
accelerated on a hybrid multicore CPU–GPU computing platform. J. Chem. Theory Comput..

[8] Li R., Sun Q., Zhang X., Chan G. K.-L. (2025). Introducing GPU Acceleration into
the Python-Based Simulations of Chemistry Framework. J. Phys. Chem. A.

[9] Guo Z., Huang Z., Chen Q., Shao J., Liu G., Pham H. Q., Huang Y., Cao C., Chen J., Lv D. (2025). ByteQC: GPU-Accelerated
Quantum Chemistry Package for Large-Scale
Systems. WIREs Comput. Mol. Sci..

[10] Storchi L., Bellentani L., Hammond J., Orlandini S., Pacifici L., Antonini N., Belpassi L. (2025). Acceleration of the
Relativistic Dirac–Kohn–Sham Method with GPU: A Pre-Exascale
Implementation of BERTHA and PyBERTHA. J. Chem.
Theory Comput..

[11] Yasuda K. (2008). Two-electron
integral evaluation on the graphics processor unit. J. Comput. Chem..

[12] Ufimtsev I. S., Martinez T. J. (2008). Quantum chemistry
on graphical processing units. 1.
Strategies for two-electron integral evaluation. J. Chem. Theory Comput..

[13] Luehr N., Ufimtsev I. S., Martinez T. J. (2011). Dynamic
precision for electron repulsion
integral evaluation on graphical processing units (GPUs). J. Chem. Theory Comput..

[14] Mazur G., Makowski M., Łazarski R. (2016). Boys function
evaluation on graphical
processing units. J. Math. Chem..

[15] Song C., Wang L.-P., Sachse T., Preiss J., Presselt M., Martínez T. J. (2015). Efficient
implementation of effective core potential
integrals and gradients on graphical processing units. J. Chem. Phys..

[16] Genovese L., Ospici M., Deutsch T., Méhaut J.-F., Neelov A., Goedecker S. (2009). Density functional
theory calculation
on many-cores hybrid central processing unit-graphic processing unit
architectures. J. Chem. Phys..

[17] Seritan S., Bannwarth C., Fales B. S., Hohenstein E. G., Isborn C. M., Kokkila-Schumacher S.
I. L., Li X., Liu F., Luehr N., Snyder J. W., Song C., Titov A. V., Ufimtsev I. S., Wang L.-P., Martínez T. J. (2021). TeraChem:
A graphical processing unit-accelerated electronic structure package
for large-scale ab initio molecular dynamics. WIREs Comput. Mol. Sci..

[18] Agarawal V., Khurana R., Liu C., Hermes M. R., Knight C., Gagliardi L. (2025). Enabling Multireference
Calculations on Multimetallic
Systems with Graphic Processing Units. J. Chem.
Theory Comput..

[19] Ufimtsev I. S., Martinez T. J. (2009). Quantum chemistry
on graphical processing units. 2.
Direct self-consistent-field implementation. J. Chem. Theory Comput..

[20] Asadchev A., Gordon M. S. (2012). New multithreaded
hybrid CPU/GPU approach to Hartree–Fock. J. Chem. Theory Comput..

[21] Tsuji S., Ito Y., Fujii H., Yokogawa N., Suzuki K., Nakano K., Parque V., Kasagi A. (2025). GPU-Accelerated Fock Matrix Computation
with Efficient Reduction. Appl. Sci..

[22] Götz, A. W. ; Wölfle, T. ; Walker, R. C. Chapter 2 - Quantum Chemistry on Graphics Processing Units; Wheeler, R. A. , Ed.; Annual Reports in Computational Chemistry; Elsevier, 2010; Vol. 6; pp 21–35.

[23] Li X., Linares M., Norman P. (2025). VeloxChem: GPU-accelerated Fock matrix
construction enabling complex polarization propagator simulations
of circular dichroism spectra of G-quadruplexes. J. Phys. Chem. A.

[24] Menczer A., van Damme M., Rask A., Huntington L., Hammond J., Xantheas S. S., Ganahl M., Legeza O. (2024). Parallel implementation
of the Density Matrix Renormalization Group method achieving a quarter
petaFLOPS performance on a single DGX-H100 GPU node. J. Chem. Theory Comput..

[25] Kriebel M. H., Tecmer P., Gałyńska M., Leszczyk A., Boguslawski K. (2024). Accelerating Pythonic coupled cluster implementations:
a comparison between CPUs and GPUs. J. Chem.
Theory Comput..

[26] Blum V., Asahi R., Autschbach J., Bannwarth C., Bihlmayer G., Blügel S., Burns L. A., Crawford T. D., Dawson W., de Jong W. A. (2024). Roadmap on methods and
software for electronic structure based simulations in chemistry and
materials. Electron. Struct..

[27] Shinde R., Filippi C., Scemama A., Jalby W. (2025). Shifting sands of hardware
and software in exascale quantum mechanical simulations. Nat. Rev. Phys..

[28] Abadi, M. ; Barham, P. ; Chen, J. ; Chen, Z. ; Davis, A. ; Dean, J. ; Devin, M. ; Ghemawat, S. ; Irving, G. ; Isard, M. ; TensorFlow: a system for Large-Scale machine learning. 12th USENIX symposium on operating systems design and implementation (OSDI 16); OSDI, 2016, 265–283.

[29] Okuta, R. ; Unno, Y. ; Nishino, D. ; Hido, S. ; Loomis, C. CuPy: A NumPy-Compatible Library for NVIDIA GPU Calculations. 31st conference on neural information processing systems 2017, 55–62.

[30] Paszke, A. ; Gross, S. ; Massa, F. ; Lerer, A. ; Bradbury, J. ; Chanan, G. ; Killeen, T. ; Lin, Z. ; Gimelshein, N. ; Antiga, L. ; Desmaison, A. ; Kopf, A. ; Yang, E. ; DeVito, Z. ; Raison, M. ; Tejani, A. ; Chilamkurthy, S. ; Steiner, B. ; Fang, L. ; Bai, J. ; Chintala, S. PyTorch: An Imperative Style, High-Performance Deep Learning Library. Advances in Neural Information Processing Systems; Curran Associates, Inc., 2019.

[31] Harris C. R., Millman K. J., van der Walt S. J., Gommers R., Virtanen P., Cournapeau D., Wieser E., Taylor J., Berg S., Smith N. J., Kern R., Picus M., Hoyer S., van Kerkwijk M. H., Brett M., Haldane A., del Río J. F., Wiebe M., Peterson P., Gérard-Marchant P., Sheppard K., Reddy T., Weckesser W., Abbasi H., Gohlke C., Oliphant T. E. (2020). Array programming
with NumPy. Nature.

[32] Focke K., De Santis M., Wolter M., Martinez
B J. A., Vallet V., Pereira Gomes A. S., Olejniczak M., Jacob C. R. (2024). Interoperable workflows by exchanging grid-based data
between quantum-chemical program packages. J.
Chem. Phys..

[33] Athavale V., Fedik N., Colglazier W., Niklasson A. M., Kulichenko M., Tretiak S. (2025). PYSEQM 2.0: Accelerated Semiempirical
Excited-State Calculations on Graphical Processing Units. J. Chem. Theory Comput..

[34] Aquilante F., Boman L., Boström J., Koch H., Lindh R., de Merás A. S., Pedersen T. B. (2011). Cholesky decomposition techniques
in electronic structure theory. Linear-Scaling
Techniques in Computational Chemistry and Physics: Methods and Applications.

[35] Pedersen T. B., Lehtola S., Fdez. Galván I., Lindh R. (2024). The versatility of
the Cholesky decomposition in electronic structure theory. WIREs Comput. Mol. Sci..

[36] Boguslawski K., Leszczyk A., Nowak A., Brzęk F., Żuchowski P. S., Kędziera D., Tecmer P. (2021). Pythonic Black-box
Electronic Structure Tool (PyBEST). An open-source Python platform
for electronic structure calculations at the interface between chemistry
and physics. Comput. Phys. Commun..

[37] Boguslawski K., Brzęk F., Chakraborty R., Cieślak K., Jahani S., Leszczyk A., Nowak A., Sujkowski E., Świerczyński J., Ahmadkhani S., Kędziera D., Kriebel M. H., Żuchowski P. S., Tecmer P. (2024). PyBEST: improved functionality and enhanced performance. Comput. Phys. Commun..

[38] NVIDIA GH200 Grace Hopper Superchip | Datasheet | 1 NVIDIA GH200 Grace Hopper Superchip,https://resources.nvidia.com/en-us-grace-cpu/grace-hopper-superchip, accessed December 10, 2025.

[39] NVIDIA Blackwell Architecture,https://www.nvidia.com/en-us/data-center/technologies/blackwell-architecture/, accessed December 10, 2025.

[40] Dongarra, J. ; Gunnels, J. ; Bayraktar, H. ; Haidar, A. ; Ernst, D. Accelerating Supercomputing: AI-Hardware-Driven Innovation for Speed and Efficiency. 2025 IEEE High Performance Extreme Computing Conference (HPEC); IEEE, 2025, pp 1–7.

[41] Uchino, Y. ; Ozaki, K. ; Imamura, T. High-Performance and Power-Efficient Emulation of Matrix Multiplication using INT8Matrix Engines. Proceedings of the SC ’25 Workshops of the International Conference for High Performance Computing, Networking, Storage and Analysis; ACM, 2025, New York, NY, USA, pp 1824–1831.

[42] Uchino Y., Ozaki K., Imamura T. (2026). Double-Precision Matrix Multiplication
Emulation via Ozaki-II Scheme with FP8 Quantization. arXiv.

[43] Brower C., Rodriguez Bernabeu S., Hammond J., Gunnels J., Xanthea S. S., Ganahl M., Menczer A., Legeza Ö. (2026). Mixed-Precision
Ab Initio Tensor Network State Methods Adapted for NVIDIA Blackwell
Technology via Emulated FP64 Arithmetic. J.
Chem. Theory Comput..

[44] Dawson W., Ozaki K., Domke J., Nakajima T. (2024). Reducing Numerical
Precision Requirements in Quantum Chemistry Calculations. J. Chem. Theory Comput..

[45] Čížek J. (1966). On the Correlation
Problem in Atomic and Molecular Systems. Calculation of Wavefunction
Components in Ursell-Type Expansion Using Quantum-Field Theoretical
Methods. J. Chem. Phys..

[46] Čížek J., Paldus J. (1971). Correlation problems in atomic and molecular systems
III. Rederivation of the coupled-pair many-electron theory using the
traditional quantum chemical methods. Int. J.
Quantum Chem..

[47] Bartlett R. J. (1981). Many-Body
Perturbation Theory and Coupled Cluster Theory for Electron Correlation
in Molecules. Annu. Rev. Phys. Chem..

[48] Bartlett R. J., Musiał M. (2007). Coupled-cluster
theory in quantum chemistry. Rev. Mod. Phys..

[49] Helgaker, T. ; Jørgensen, P. ; Olsen, J. Molecular electronic-structure Theory; Wiley: New York, 2000.

[50] Shavitt, I. ; Bertlett, R. J. Many-Body Methods in Chemistry and Physics. MBPT and Cloupled-Cluster Theory; Cambridge University Press, 2009; Vol. 2; 978-0–511–59643–8.

[51] Koch H., Sánchez de Merás A., Pedersen T. B. (2003). Reduced scaling
in electronic structure calculations using Cholesky decompositions. J. Chem. Phys..

[52] Wrocław Centre for Networking and Supercomputing,https://wcss.pl/en/, accessed December 10, 2025.

[53] Cyfronet Technical Documentation,https://docs.hpc.cyfronet.pl/, accessed December 10, 2025.

[54] Dunning T. H. (1989). Gaussian basis sets for use in correlated molecular
calculations. I. The atoms boron through neon and hydrogen. J. Chem. Phys..

[55] Ditchfield R., Hehre W. J., Pople J. A. (1971). Self-consistent molecular-orbital
methods. IX. An extended Gaussian-type basis for molecular-orbital
studies of organic molecules. J. Chem. Phys..

[56] Hehre W. J., Ditchfield R., Pople J. A. (1972). Self-consistent molecular orbital
methods. XII. Further extensions of Gaussian-type basis sets for use
in molecular orbital studies of organic molecules. J. Chem. Phys..

[57] Hariharan P. C., Pople J. A. (1973). The influence
of polarization functions on molecular
orbital hydrogenation energies. Theor. Chim.
Acta.

[58] Kendall R. A., Dunning T. H., Harrison R. J. (1992). Electron-affinities
of the 1st-row
atoms revisited - systematic basis-sets and wave-functions. J. Chem. Phys..

[59] Szczuczko L., Boguslawski K. (2025). A Cross-Platform
Graphical User Interface Using Web
Technologies: Simplifying the Setup for PyBEST Calculations. Int. J. Quantum Chem..

[60] Shen T., Zhu Z., Zhang I. Y., Scheffler M. (2019). Massive-Parallel Implementation of
the Resolution-of-Identity Coupled-Cluster Approaches in the Numeric
Atom-Centered Orbital Framework for Molecular Systems. J. Chem. Theory Comput..

[61] Fales B. S., Curtis E. R., Johnson K. G., Lahana D., Seritan S., Wang Y., Weir H., Martínez T. J., Hohenstein E. G. (2020). Performance of coupled-cluster singles and doubles
on modern stream processing architectures. J.
Chem. Theory Comput..

