# Supplementary material for: Efficient Coupled-Cluster Python Frameworks for Next-Generation GPUs: A Comparative Study of CuPy and PyTorch on the Hopper and Grace Hopper Architecture
Source: J Chem Theory Comput. 2026 Jul 2;22(13):6533–46. doi: 10.1021/acs.jctc.6c00558 (PMC13374028; doi:10.1021/acs.jctc.6c00558)
Supplement: Supplementary file 1 [file ct6c00558_si_001.pdf]

# Efficient Coupled-Cluster Python Frameworks for Next-Generation GPUs: A Comparative Study of CuPy and PyTorch on the Hopper and Grace Hopper Architecture

Antonina Dobrowolska[1], Julian Świerczyński[1], Paweł Tecmer[1], Emil Sujkowski[1], Somayeh Ahmadkhani[1,2], Grzegorz Mazur[3], Klemens Noga[4], Jeff Hammond[5], and Katharina Boguslawski[1,*]

[1] *Institute of Physics, Faculty of Physics, Astronomy and Informatics, Nicolaus Copernicus University in Torun, Grudziadzka 5, 87-100 Torun, Poland*
[2] *Present address: Department of Mathematics and Computer Science, Freie Universität in Berlin, Germany*
[3] *Department of Computational Methods in Chemistry, Jagiellonian University, Faculty of Chemistry, Jagiellonian University, Gronostajowa 2, 30-387 Kraków, Poland*
[4] *Academic Computer Centre Cyfronet AGH, Nawojki 11a, 30-950 Kraków, Poland*
[5] *NVIDIA Helsinki Oy, 00180 Helsinki, Finland*

[*] Corresponding author: k.boguslawski@umk.pl

## Supplementary Information

# Pseudo-code snippet S1: the C-split procedure

```
# parts_a, parts_b, parts_c correspond to number of batches we calculated in axes 'a
    ', 'b', 'c' respectively
# Second Cholesky array is split along axis b (x[b]d)
STORE b—batched parts of args[1] as views in cl2
FOR x in parts_b
    APPEND to chol_chunk_lengths_2 size of batched b axis cl2[x]
ENDFOR
# Example: xac,xbd,ecfd —> efab we split:
# [a][b][c]d, e[c]fd —> ef[a][b]
# First dense array args[2] is split along axis c (e[c]fd)
STORE c—batched parts of args[2] as views in operand
# Next, first Cholesky vector is split along axis c (xa[c])
STORE c—batched parts of args[0] as views in cl1

FOR c in parts_c
    MOVE c—th operand view to the GPU and store as operand2
    # Next, first Cholesky vector is split along axis a (x[a][c])
    MOVE c—th view of a—batched cl1 to the GPU and store as chol_1
    FOR x in parts_a
        APPEND to chol_chunk_lengths_1 size of batched a axis chol_1[x]
    ENDFOR
    SET start_b as the beginning of the view of the part of the matrix we copy to
   VRAM in axis `b`
    SET end_b as the ending of the view of the part of the matrix we copy to VRAM in
    axis `b`
    FOR b in parts_b
        ADD chol_chunk_lengths_2[b] to end_b
        SET start_a as the beginning of the view of the part of the matrix we copy
   to VRAM in axis `a`
        SET end_a as the ending of the view of the part of the matrix we copy to
   VRAM in axis `a`
        MOVE b—th view of cl2 to the GPU and store as chol_2
        FOR a in parts_a
            ADD chol_chunk_lengths_1[a] to end_a
            COMPUTE tensor contraction using tensordot between asarray_gpu(chol_1[a
   ]) and chol_2 along axes=(0, 0) and store in result_temp
            COMPUTE tensor contraction using tensordot between result_temp and
   operand2 along axes=([1, 3], [axis_c, axis_d]) and store in result_temp_2

            DELETE result_temp and clean VRAM
            TRANSPOSE result_temp_2 if need and store as result_part

            DELETE result_temp_2 and clean VRAM

            MOVE result_part to CPU and add to view of final result array defined by
    start_a, start_b, end_a and end_b

            DELETE result_part and clean VRAM
            SET start_a as end_a
        ENDFOR
        SET start_b as end_b
    ENDFOR
```

```
ENDFOR
DELETE cl1, cl2, operand2 and clean VRAM
```

## Pseudo-code snippet S2: the generic batching procedure

```
# Generic GPU helper function performing tensor contractions with tensordot
# Args:
#   subscripts (str): einstein summation label, e.g.,s 'xac,xbd,defc->abfe'
#   operands (np.ndarray): first and second are arrays of Cholesky or dense type
# Returns:
#   np.ndarray: the final output of a tensor contraction

# Sanity check
CHECK subscripts for proper shape

# If possible split subscript into input and output scripts
GET inscripts and outscript from subscripts

# Deduce optimal path for tensordot
GET path from subscripts and operands using np.einsum_path or return a list of
    tuples

SET mempool to available VRAM

SET op0 to operand[0] and op1 to operand[1]

# Get number of batches for op0 and op1, the corresponding axes (the ones
# that are split/batched), and the label (ind) of the splitted subscripts
# (abcd...)
SET n_batch_0, axis0, ind0, n_batch_1, axis1, ind1 to batch size, axis, and the
    label (a, b, ..) to be batched of op0 and op1

# We will batch the first arrays that appear in einsum_path and in the output
# They correspond to operands[step0[0]] and operands[step0[1]]
SET step to path[1]

# Split first two operands contained in first step of path into
# batches under the condition that input and output share axis indices
TAKE op0 as the step[0]-th element of operands list and split it into n_batch_0
    along axis axis0
STORE batched array as a view in op0_batched
TAKE op1 as the (step[1]-1)-th element of operands list and split it into n_batch_1
    along axis axis1
STORE batched array as a view in op1_batched

# Loop over batched operators
# start_X and end_X indicate the view of the batched array
FOR op0_ in op0_batched
     # Update view of results array for axis0
     # The batched scripts need to show up in the results, otherwise
     # they are simply ignored and the whole array is taken as view
```

```
    SET view for axis0 going from first to last index of the current batch

    FOR op1_ in op1_batched
        # Update view of results array for axis1
        SET view for axis1 going from first to last index of the current batch
        # Copy subscripts as they get deleted during the batching process
        COPY scripts into scripts_
        # Create a deep copy of the path. Due to batching, we will
        # execute the same path several times
        # Each path gets deleted AFTER it has been executed
        COPY path into path_
        # Create a shallow copy (not copying data) of operand list
        # We loop through the list for each batched view
        # In each iteration, the operands are popped (deleted) from
        # the list. A copy is needed to restart the loop in each
        # batched step
        SHALLOW COPY the list of operands to operands_

        # Loop over all steps in the contraction path
        For step in path_
            # For the 0-th iteration, we need to take the batched arrays
            # For each subsequent path step, we need to pop the next operand
            # contained in the list of operands.
            # The (copied) list of operands contains only unused arrays, that
            # is arrays that have NOT been contracted yet
            POP op0 from operands[step[0]] or take op0_ in first iteration
            POP op1 from operands[step[1]-1] or take op1_ in first iteration
            # Get first subscripts used for op0 (the first in the list of
            # operands)
            POP script0 from scripts_[step[0]]
            # Get view of new op0 for first batched index (ind0)
            # We need to check if ind0 or ind1 are contained in script0
            # If yes, we need to adjust the view, otherwise the take the
            # whole axis
            UPDATE view_0 for batched index ind0
            IF ind1 is in script0 THEN
                UPDATE view_0 for batched index ind1
            ENDIF
            # Update view of op0 so that dimensions matched
            UPDATE view of op0 to match dimension as op0[tuple(view_0)]
            # Subscripts for second operand
            POP script1 from scripts_[step[1] - 1]
            # Find summation axes used in tensordot notation
            FIND axes that are summed over for op0_ and op1_ using tensordot and
  store them in axis_
            # Find default outscript of tensordot operation
            # It may differ from the outscript of the overal tensor
            # contraction due to the way tensordot works
            # Required for transposition step below
            UPDATE outscript as tensordot may change the order of the axes
            # Move arrays to GPU
            MOVE batched op0 to GPU and store as op0_gpu
            MOVE batched op1 to GPU and store as op1_gpu
            # Do contraction on GPU (finally)
```

```
            COMPUTE tensor contraction using tensordot between op0_gpu and op1_gpu
    along axes=axis_ and store in outmat
            # Cleanup
            DELETE op0_gpu and op1_gpu and clear VRAM
            # Update the list of operands and subscripts
            # The partially contracted array and its subscripts
            # (here outscript_) are appended to the working copies
            # of the operands and scripts. They will be used in the
            # next contraction step
            APPEND outmat to operands_ for next contraction step contained in path_
            APPEND next contraction recipe to scripts_
        ENDFOR
        # Do transposition if required
        IF output array needs to be transposed THEN
            TRANSPOSE outmat
        ENDIF
        # Add batched contraction result to view of result array
        MOVE outmat to CPU and add to view of final result array
        DELETE outmat and clear VRAM
        ENDFOR
    ENDFOR
ENDFOR
```
